# Open‐label long‐term treatment of add‐on triheptanoin in adults with drug‐resistant epilepsy

**DOI:** 10.1002/epi4.12391

**Published:** 2020-04-12

**Authors:** Karin Borges, Neha Kaul, Jack Germaine, Catalina Carrasco‐Pozo, Patrick Kwan, Terence J. O’Brien

**Affiliations:** ^1^ School of Biomedical Sciences Faculty of Medicine The University of Queensland St. Lucia QLD Australia; ^2^ Department of Allied Health (Clinical Nutrition) Royal Melbourne Hospital University of Melbourne Parkville Vic Australia; ^3^ Departments of Medicine and Neurology Royal Melbourne Hospital University of Melbourne Parkville Vic Australia; ^4^ Departments of Neuroscience and Neurology The Central Clinical School Monash University and The Alfred Hospital Melbourne Vic Australia; ^5^ Discovery Biology Griffith Institute for Drug Discovery Griffith University Nathan QLD Australia

**Keywords:** anaplerosis, focal seizure, medium‐chain fatty acid, tca cycle

## Abstract

**Objective:**

To investigate feasibility, safety, and tolerability of long‐term (48 weeks) add‐on treatment with triheptanoin (UX007), the triglyceride of heptanoate, in adults with drug‐resistant epilepsy.

**Methods:**

This extension study was offered to adult participants with drug‐resistant epilepsy who completed a 12‐week randomized controlled trial of add‐on medium‐chain triglycerides (MCT) vs triheptanoin. Participants were asked to titrate triheptanoin to their maximum tolerated dose over 3 weeks, followed by 48‐week maintenance before tapering or treatment extension. The primary aims were to assess retention and safety of the triheptanoin treatment, and secondary aims to assess the tolerated doses and changes in seizure frequency.

**Results:**

Eleven adults were enrolled and ten people were analyzed (because one patient was diagnosed as having nonepileptic seizures while on the study). Two adults finished the study and extended their treatment. Eight participants withdrew from the study, due to lack of efficacy (n = 3), unknown reasons (n = 2), belief of weight gain (n = 1), wanting to try a different treatment (n = 1), and a colonoscopy (n = 1). Diarrhea in two people and bloating in one person were deemed possibly related to treatment, but other adverse events were not. The duration of maintenance treatment dose was 27‐513 days (median 247 days, range 27‐513 days), and 0.49 ‐1.1 mL/kg triheptanoin was taken per day (0.77 ± 0.19 mL/kg, mean ± standard deviation, 40‐100 mL/d). Two participants experienced >90% and three people >50% reduction in seizure frequency, and all had focal seizures. The median seizure reduction was 48% (average 38%).

**Significance:**

Our results indicate antiseizure effects of triheptanoin on focal seizures in 5 out of 10 adults. However, only two people finished and extended the 48‐week add‐on treatment phase, despite lack of safety or tolerability issues.

More studies focused on improved treatment formulations, the potential of lower dosages, and efficacy are needed. Trial registration number: ACTRN12615000406505.

**Key Point**

- Triheptanoin add‐on treatment (0.77 ± 0.19 mL/kg, 40‐100 mL/d) was tolerated for 27‐513 days (average 253 days).
- Only 2 out of 10 people finished the 48‐week treatment
- Diarrhea in two people and bloating in one person were adverse effects deemed possibly related to treatment.
- Five out of 10 people, all with focal seizures, showed >50% reduction in seizure frequency (mean 48% seizure reduction).
- Further trials are suggested to focus on improved treatment formulations and efficacy

## 1 INTRODUCTION

**FIGURE 1:**
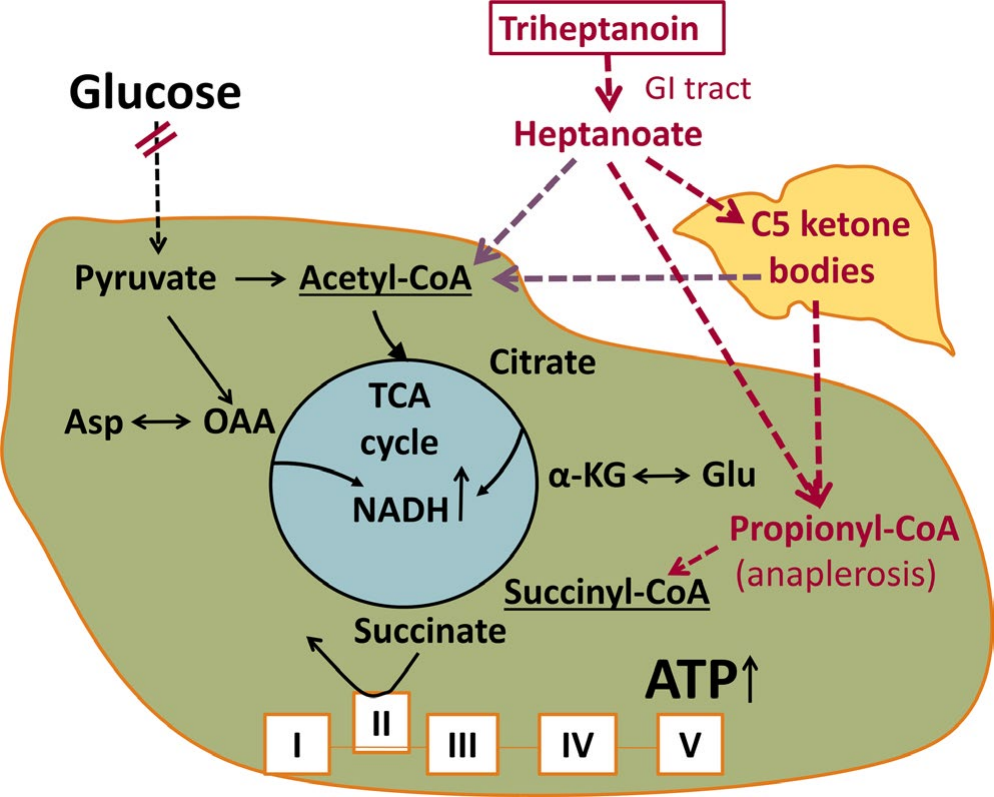
Diagram of the proposed biochemical effects of triheptanoin in epilepsy. The main fuel of the brain is usually glucose. Entry of glucose‐derived carbons into the TCA cycle produces most of the ATP via oxidative phosphorylation, but also precursors for lipids and amino acids, such as aspartate and glutamate. The red double lines indicate that in many epilepsy types, FDG‐PET indicates impaired glucose metabolism in epileptogenic areas. There is also evidence for shortages of TCA cycle intermediates. Both these impairments can result in local shortages of ATP as well as carbons to produce lipids from citrate and amino acids (e.g. glutamate and aspartate). Together this may contribute to dysregulation of neuronal signaling and subsequent seizure generation. Auxiliary sources of carbons for the brain can be provided by triheptanoin, which is metabolized to the medium‐chain fatty acid heptanoate and C5 ketone bodies,  which are further metabolized to auxiliary acetyl‐CoA and propionyl‐CoA. The latter can be carboxylated to succinyl‐CoA, thereby refilling the TCA with C4 intermediates (anaplerosis). This is important to compensate for the loss of carbons from the TCA cycle for production of lipids and amino acids and to continue efficient TCA cycling

Heightened excitability of the brain and reduced glucose metabolism in epileptogenic areas are hallmarks of epilepsy in people and animal models.^1^, ^2^, ^3^, ^4^, ^5^ Reduced glucose metabolism could lead to local energy deficiency and is likely to contribute to seizure generation.^6^ In addition, a shortage of glucose‐derived carbons and their metabolites can occur, which includes amino acids and lipids, vital for regulated neuronal signaling (Figure 1). The decreases in brain levels of glutamate, glutamine, and malate found in people with epilepsy and rodent chronic epilepsy models indicate that the tricarboxylic acid (TCA) cycle is deficient of intermediates containing 4 or 5 carbons.^7^, ^8^, ^9^, ^10^, ^11^ This is expected to reduce the flux through the TCA cycle and the production of ATP, amino acids, and lipids produced from the cycle intermediates. Thus, refilling of the pools of C5 and C4 TCA cycle metabolites (anaplerosis) is needed in addition to energy.^5^, ^11^ Auxiliary sources of carbons for the brain, such as medium‐chain fatty acids or ketone bodies, can address reduced glucose and energy metabolism in epilepsy (reviewed by Refs 5, 12, Figure 1). Furthermore, triheptanoin, the triglyceride of the medium‐chain fatty acid heptanoate, is also anaplerotic by providing heptanoate and the C5 ketone bodies β‐ketopentanoate and β‐hydroxypentanoate, which can enter the Krebs cycle as succinyl‐ and acetyl‐CoA (Figure 1).^5^, ^13^, ^14^, ^15^, ^16^, ^17^ Originally, triheptanoin has been used in various metabolic disorders,^5^, ^18^, ^19^, ^20^, ^21^ including glucose transporter 1 deficiency which commonly includes seizures.^18^, ^22^, ^23^ In epilepsy models, providing 35% of energy intake of triheptanoin has shown anticonvulsant, beneficial metabolic, and antioxidant effects.^5^, ^9^, ^17^, ^18^, ^24^, ^25^, ^26^ Also, an open‐label study evaluating the effects of triheptanoin in children with drug‐resistant epilepsy found the treatment to be tolerated in 8 out 12, with more than 50% seizure reduction in 5 out of 8 children, including one child who became seizure‐free for 30 weeks.^27^

In a previous randomized controlled trial (RCT),^28^ we investigated safety and tolerability of triheptanoin vs medium‐chain triglycerides (MCT, which contain triglycerides with octanoate and decanoate) as add‐on treatments in adults with drug‐resistant epilepsy. Patients who had completed the study were invited to take part in this long‐term open‐label extension trial of triheptanoin at maximal tolerated dose to assess retention and safety of the triheptanoin treatment, and secondary aims to assess the tolerated doses and changes in seizure frequency. Participants were advised to mix triheptanoin with food and to reduce energy intake of regular foods (but no specific types of macronutrients) to avoid gastrointestinal problems and excess energy intake.

## 2 METHODS

### 2.1 Classification of evidence

We sought to investigate whether long‐term treatment with triheptanoin is safe and tolerated as an add‐on therapy in people with drug‐resistant epilepsy. The evidence generated from this trial is classified as class IV, because of the relatively small subject numbers and open‐label treatment.

### 2.2 Standard protocol approvals, registrations, and patient consents

We confirm that we have read the Journal's position on issues involved in ethical publication and affirm that this report is consistent with those guidelines. Ethics approval was granted by the Human Research and Ethics Committees of Melbourne Health and the University of Queensland. Written informed consent was provided by all participants or their legal guardians, prior to any study procedures being undertaken. The trial was registered with the Australian New Zealand Clinical Trials Registry (registry no: ACTRN12615000406505). The trial protocol will be made available by the corresponding author upon reasonable request.

### 2.3 Organization of study

The study was a single‐center, open‐label trial. The study was conducted from 2015 to 2016 at the Royal Melbourne Hospital, Victoria, Australia, and was monitored for safety by an independent Clinical Research Organisation (CRO), Neuroscience Trials Australia Inc.

### 2.4 Participants

Only participants who finished the previous 12‐week trial of add‐on medium‐chain triglycerides vs triheptanoin^28^ were allowed to join this trial. The previous trial included male or female subjects (≥18 years old) with epilepsy who had failed treatment with ≥2 antiseizure drugs and experienced at least 2 seizures of an eligible type per 28 days over two months prior to enrollment despite treatment with at least one antiseizure drug at clinically appropriate doses. Eligible seizure types included focal unaware, focal to bilateral tonic seizures, focal aware motor, generalized tonic, and tonic and atonic seizures.^29^ Exclusion criteria were eating and psychiatric disorders, substance abuse, currently on ketogenic diet, nonepileptic seizures, changes of antiseizure medication 1 month before trial, pregnancy, and breastfeeding.

Some participants were enrolled immediately after completion of the first trial, while others had a long gap period between 154 and 638 days ([median: 348 days], see Figures S1,S2).

### 2.5 Study intervention

All participants received add‐on UX007 (triheptanoin, Ultragenyx Pharmaceuticals Inc) in oil form. After dietitian counseling, energy intake was calculated from 4‐day food diaries provided using Foodworks 7 (Xyris Software). Patients were then given a titration scheme to increase their treatment dose over a period of three weeks to their maximum tolerated dose of maximal 35% of energy intake or 100 mL/d. Participants reported all treatment doses taken in their treatment diaries, and the intake was verified during study visits. The treatment period consisted of a 3‐week on‐titration phase followed by a 48‐week maintenance phase with study visits every 12 weeks. Then, participants titrated off the treatment over 3 weeks and remained off the treatment for 4 weeks, until the last study visit. Participants with seizure reduction were given the option to continue treatment under a compassionate access scheme without downtitration.

### 2.6 Outcome measures

The study's primary prespecified end points were study retention, measured as the number and proportion of participants taking treatment over the 48‐week treatment period, and safety, measured as the number of adverse events that are causally (defined as probably and definitely) related to study intervention over the on‐titration and maintenance treatment periods. Due to the low number of finishing participants, the secondary end points were changed to include all participants to find the tolerated dose per day, as measured as the average treatment dose actually taken over the maintenance treatment period. The responder rate was calculated as the proportion of participants who showed ≥50% improvement in seizure frequency during their maintenance phase as compared to the original baseline from the previous study (see Figures S1 and S2).

### 2.7 Study procedures

**FIGURE 2:**
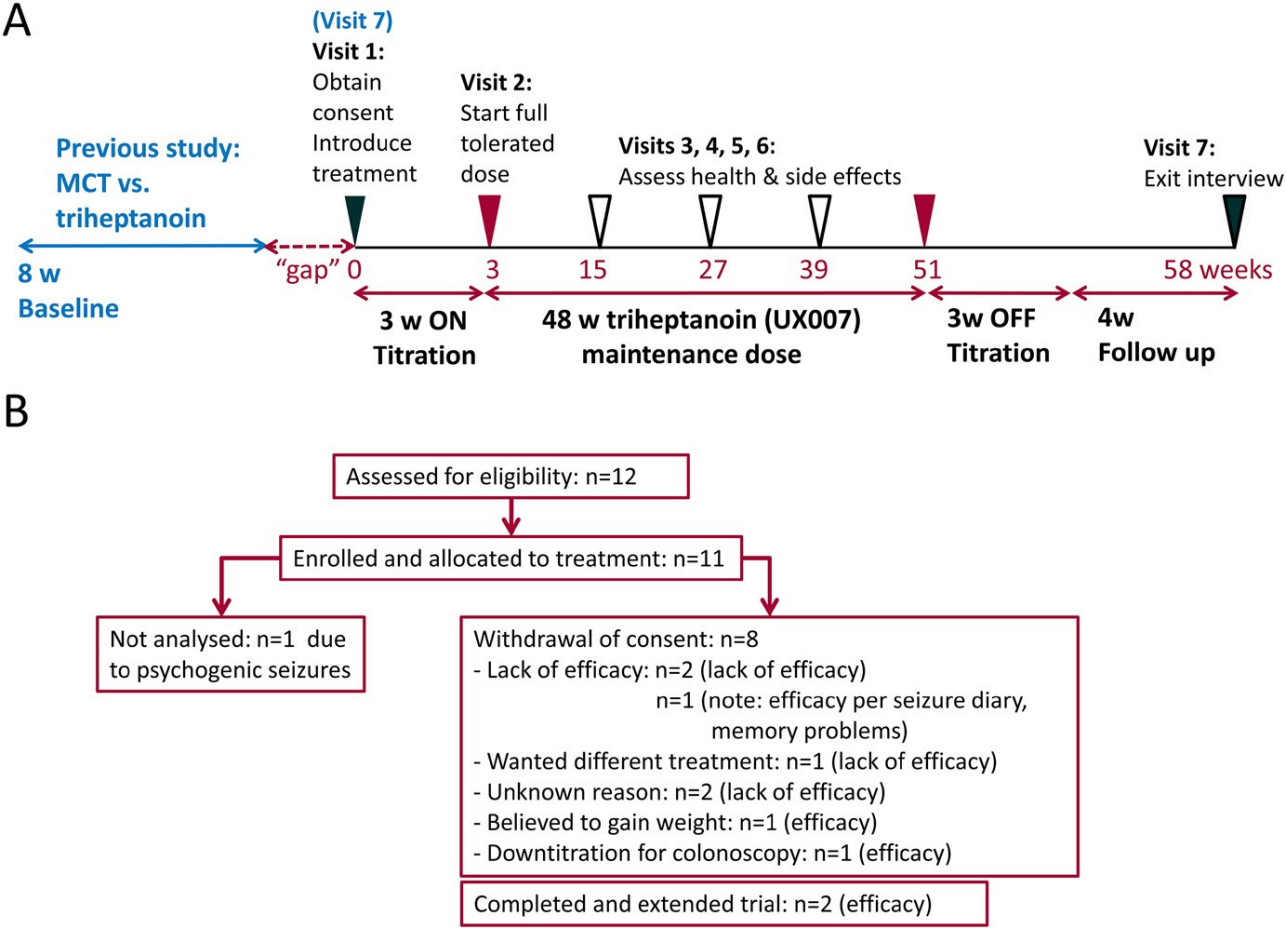
Schematics specifying the trial design (A) and flow diagram indicating the number of people with refractory epilepsy enrolled until study completion (B). A, Clinic visits are indicated as triangles on the weekly timeline. Five patients directly moved at their last visit (7) of the randomized study into this study; five other people had a gap period. The seizure frequencies of the 8‐week baseline period from the previous study were used for comparisons to those during the maintenance period, as shown in Figures S1 and S2. The on‐titration period for triheptanoin add‐on treatment was 3 wk until maximal tolerated dose was reached and was maintained for the 48‐week maintenance phase. Then, triheptanoin was titrated off for 3 wk and followed by a four‐week period without add‐on treatment. B, The diagram shows the number of participants analyzed for outcomes and reasons for withdrawal from the study.

After giving informed consent, participants had a medical history taken, a physical and neurological examination, and an evaluation of inclusion and exclusion criteria. Blood and urine were analyzed for acyl‐carnitines and organic acids, respectively, by mass spectroscopy to exclude metabolic abnormalities. Also, serum chemistry, blood cell counts, and when applicable the levels of valproate, carbamazepine, and phenytoin were evaluated at all hospital visits before and after the treatment and every twelve weeks during treatment (Figure 2A). Participants were instructed on the keeping of daily seizure diaries and 4‐day food diaries and were not allowed to change their antiseizure medications during the study period. At all visits, a dietitian counseled patients individually on healthy eating, as per Australian dietary guidelines (2013). Also, she provided advice on how to optimize the remaining diet for adequate nutrition and minimization of gastrointestinal side effects or body weight changes. Participants were given an Australian Guide to Healthy Eating, and we recommended to serve a small portion of a meal and then add the recommended dose of the oil. If still hungry once finished, more food without oil could be eaten. No specific recommendations were given regarding total caloric intake.

On visits 2, 4, 6, and 7, participants were asked to fill out the QOLIE‐89^30^, ^31^ questionnaire, the Liverpool Adverse Events Profile (LAEP)^32^ and the Hospital Anxiety and Depression Scale (HADS). Adverse events were noted throughout the study and assessed regarding causality to treatment. All severe adverse events (SAEs) were reviewed by the study CRO. Both the site investigator and the sponsor of the study (University of Queensland) assessed the potential relationship between SAEs and study drug.

### 2.8 Sample size and statistical analysis

Data from all participants were included, even if the full treatment period was not completed (Figure 2B, Figures S1, S2). We report median and interquartile ranges as well as averages and standard deviations. The questionnaires were analyzed using a Kruskal‐Wallis test. All statistical comparisons were performed using excel or GraphPad prism version 7.0 (GraphPad Software).

## 3 RESULTS

### 3.1 Patient cohort, retention, treatment duration, maximal tolerated dose, and safety

**FIGURE 3:**
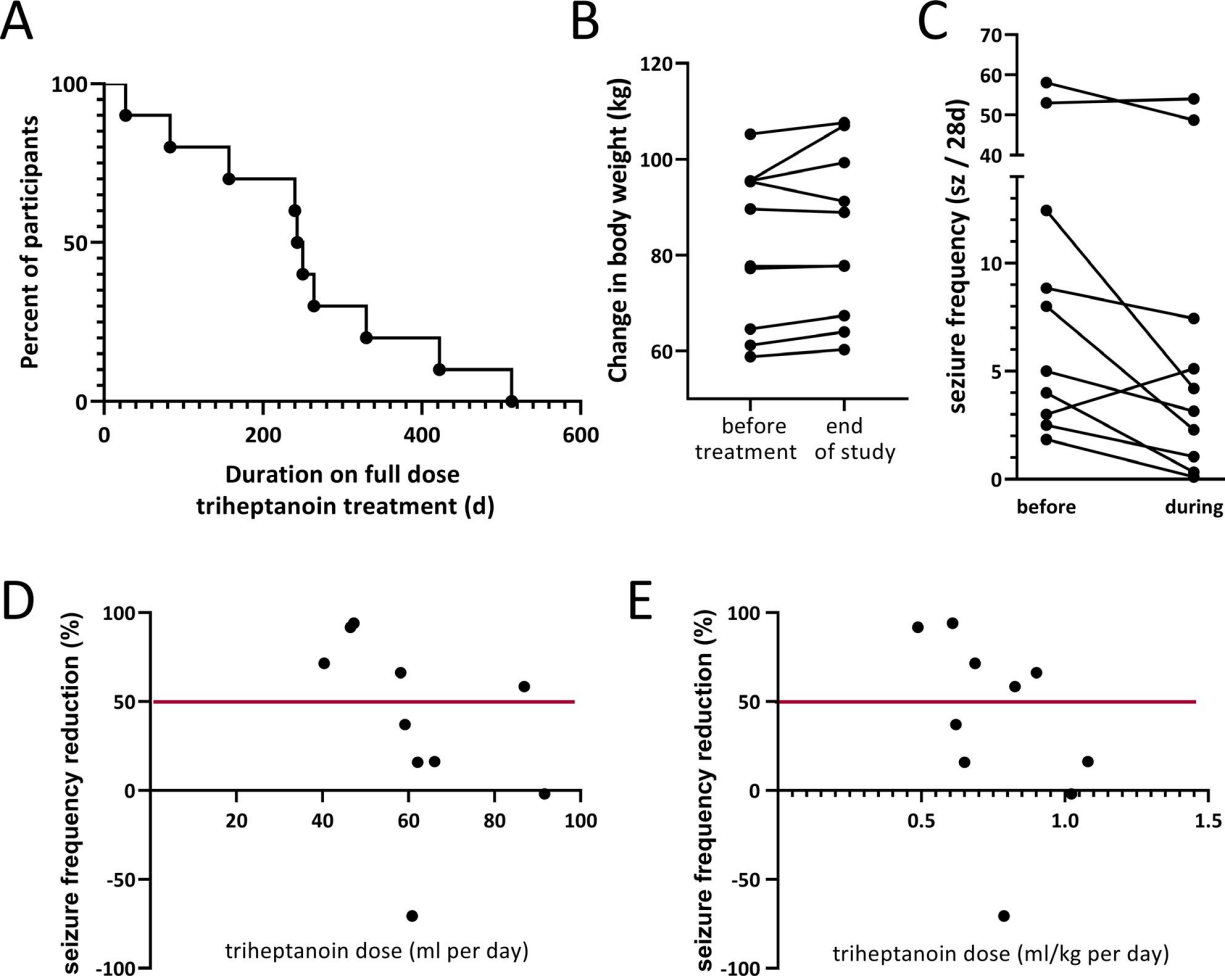
A, Duration on full treatment dose and B the changes in body weight are shown for all participants. B, The changes in body weight (kg) from the beginning of the study until the end of the triheptanoin treatment vs until the end of the full study (after downtitration and no add‐on treatment for 4 wk) are shown for each participant. Please note that several participants lost some of the body weight gained at the end of the maintenance phase. C, The changes in seizure frequencies (number of seizures/28 d) from baseline vs maintenance treatment phases are shown. D,E There is no relationship of changes in seizure frequency vs the volume of triheptanoin taken (D) or the dose taken relative to body weight (E).

Figure 2B shows the participant flow and Figures S1 and S2 show overviews of body weights, doses, and seizure frequencies over time for each participant. Eleven people were enrolled, but one individual was excluded as diagnosed to have psychogenic nonepileptic seizures after enrollment. The characteristics of the participant population included and analyzed are shown in Table 1. Five people were immediately enrolled at their last visit of the previous study, while another five people were enrolled after a large “gap” period ranging from 154‐638 days (median 348 days). The proportion of participants remaining on triheptanoin for 48 weeks was 20%. (Table 2, Figures 2B and 3A).

**TABLE 1:** Demographics and characteristics

	N = 10
Age (years), mean (SD)	45.3 (10.9)
Gender	
Female, n (%)	4 (40%)
Male, n (%)	6 (60%)
Body weight (kg), mean (SD)	82.1 (16.5)
Body mass index, mean (SD)	28.2 (6.3)
Epilepsy etiology, n (%)	
Temporal	3 (30%)
Extra temporal or unknown	7 (70%)
Seizure types, n (%)	
Focal unaware	9 (90%)
Focal to bilateral tonic clonic	3 (30%)
Focal aware motor	1 (10%)
Generalized tonic clonic	0
Prior neurological surgery, n (%)	
None	7 (70%)
1 (tumor resection, temporal lobectomy)	2 (20%)
2 (temporal lobectomy and hemispherectomy)	1 (10%)
Number of concomitant AEDs, n (%)	
2	1 (10%)
3	3 (30%)
4	6 (60%)
Seizures per 28 d, median (IQR)	4.9 (3.8, 22.2)
Caloric (Kcal) intake per day, median (IQR)	1735 (1285, 2775)

**TABLE 2:** General treatment effects

	Triheptanoin
Proportion of patients completing trial	2/10 (20%)
Number of participants extending treatment Proportion of patients with adverse events	2 8/10 (80%)
Total number of adverse events possibly or likely related to treatment	3 (n = 3)
Maintenance period (days, median, IQR)	247 (139, 353)
Dose taken during maintenance period (median, IQR)	
Volume of treatment/day (mL)	60 (47, 83)
Treatment dose/ body weight (mL/kg)	0.74 (0.62, 0.93)
	
Changes in body weight from v1 to end of treatment (kg)	1.9 (1.1, 2.8)
Changes in BMI from v1 to end of treatment (kg/m^2^)	0.74 (0.37, 1.75)
	
Percentage (median, IQR) of baseline seizures during treatment period (95% CIs)	58.3 (21.5, 102) (−2,92)
Responder rate (>90% seizure reduction)	2 (20%)
Responder rate (>50% seizure reduction)	3 (30%)

The duration on the maintenance treatment dose was 27‐513 days (median 247 days, average 253 ± 146 days, mean ± standard deviation, Figure 3A, Table 2). This includes two people with seizure frequency reductions who extended their treatments without downtitration to a total of 422 and 513 days. Eight participants withdrew their consent before the end of the study (Figure 2B), specifically due to lack of efficacy (n = 3, with one person with memory issues who actually showed a 72% reduction in seizure frequency in their seizure diary), unknown reasons (n = 2), believing to gain weight, despite loss of 2 kg from 95.3 to 93.3 kg, and 92% reduction in seizure numbers during the 25‐week treatment, after which she revoked consent (n = 1), wanting to try a different treatment (n = 1, there was a 70% increase in seizure numbers), and a need for a colonoscopy (n = 1). During the maintenance phase, participants took 0.49‐1.1 mL/kg triheptanoin per day (average 0.77 ± 0.19 mL/kg, 40‐100 mL/d, n = 10, Table 2).

### 3.2 Adverse events and body weight changes during treatment

None of the blood tests, including lipid profiles, showed any clinically significant changes during the treatment period. The proportion of people with adverse events was 80% (Table 2). There were 3 nonserious adverse events that were determined as probably related to treatment, namely diarrhea in 2 people and bloating in one person. All other adverse events appeared to not be related, including moderate urine tract infections (n = 2). In addition, one person suffered two serious adverse events during the extension phase, namely increases in seizure activity and stent placement.

Participants had body weights of 58.8 to 105.2 kg (median 84 kg) with a BMI of 20.8 to 37.3 kg/m^2^ (median 26.5) at the beginning of the study (Table 1). The changes in body weight ranged from a loss of 2.0 kg to a gain of 10.3 kg (median 1.9 kg, IQR 1., 2.8 kg; mean 2.4 kg, standard deviation 3.1 kg, body weight changes (kg) relative to BMI: −0.05 to 0.28, median 0.065, mean 0.86) over the up to 48‐week treatment period. The person with 10 kg weight gain and their caregivers were specifically counseled to improve their diet and at 40 weeks to stop the trial (Figure S2E). The caregivers continued with treatment and withdrew consent one week later without providing any reason. Please note that this participant, who had a temporal lobectomy and later hemispherectomy, had 45% reduction in the first blinded trial (from 8.8 seizure per 4 weeks) and seizures more than doubled after off titration. Thereafter, triheptanoin reduced their spike in seizure frequency by 47%. Half of the participants lost their gained weight during off titration of triheptanoin and the triheptanoin‐free period (Figure 3B). At the end of the study compared to the beginning, we found a loss of 4.1 kg to a gain of 11.4 kg (median 2 kg, IQR −0.2, 3.1; mean 2.1 kg, standard deviation 4.0 kg. The changes in BMI over the treatment period ranged from a loss of 0.76 to increase of 4.7 kg/m^2^ (median 0.74, mean 1.3, standard deviation 1.7 kg/m^2^, Table 2). There were no changes in the LAEPD or the QOLIE‐89 scores during the time of treatment relative to those at the first study visit (Kruskal‐Wallis tests, Figures S3, S4). Too few participants completed the Hospital Anxiety and Depression Scale (HADS) to be meaningfully analyzed.

### 3.3 Seizures

The changes in seizure frequency ranged from 94% seizure reduction to an increase by 70%, with a median of 47.7% seizure reduction (11.4 to 76.6%, interquartile ranges, Table 2, Figure 3C). Efficacy with over 90% reduction in seizure frequency was found in two people, including the person, who withdrew from the study because of belief of weight gain, but showed 92% reduction (Figure S1D). Another person achieved 94% reduction of seizures over their full maintenance phase of 248 days with seizure freedom for 167 days (Figure S1E), but needed to reduce the dose for a colonoscopy near the end of the full treatment period. There were three adults with over 50% reduction, including the two participants who extended their treatment (Figure S1A,B) and one person who withdrew because of a perception of lack of efficacy, who also had memory problems (72% seizure reduction, Figure S1C). All these five participants had focal seizures, and four specially had focal unaware seizures. Please note that one person suffered a 70% increase in seizures from 3 to 5 seizures per 28‐day period (Figure S2B).

Interestingly, the five participants who experienced no efficacy in this study also showed no effect with triheptanoin or MCT in our previous study (Figure S2). On the other hand, two people who previously had no reduction with MCT and triheptanoin, respectively, showed 58% and 72% reduction in this open‐label extension trial (Figure S1B,C). In addition, three out of the people with > 50% reduction in seizures also had >50% reduction in seizures in the previous trial, two with MCT and one with triheptanoin treatment (Figure S1A,D,E).

### 3.4 Dose vs antiseizure effects

In the people with seizure control, the effective triheptanoin dose was between 40 and 89 mL/d (median 47 mL) or 0.49 and 0.83 mL/kg body weight (median 0.69 mL/kg, Figure S1). There were no correlations of seizure control to the dose taken (Figure 3D,E).

## 4 DISCUSSION

This is the first open‐label study of long‐term add‐on triheptanoin (UX007) in adults with drug‐resistant epilepsy. The main findings were the following: (a) most of the participants (eight of ten) did not finish the study; (b) on the other hand, the 10 participants were able to consume 0.49 ‐1.1 mL/kg triheptanoin per day (0.77 ± 0.19 mL/kg, 40‐100 mL/d) for a duration of 27‐513 days (median 247 days, 253 ± 146 days mean ± standard deviation) and nobody withdrew from the study due to problems with tolerability or safety; (c) there were no serious adverse effects that were thought to be causally related to the treatment. The main side effects were diarrhea and bloating. Except for one person who gained more than 10% of his body weight, body weight changes were not of clinical significance, (d) two participants experienced >90% and three  participants >50% reductions in seizure frequency during full‐dose treatment (50% of trial particpants). All these participants had focal seizures, mostly with loss of awareness.

### 4.1 Feasibility and tolerability

Ten eligible participants were enrolled into this open‐label long‐term follow‐up study of the previous 34 enrolled in the previous randomized blinded RCT of MCT vs triheptanoin.^28^ Only 2 out of 10 patients completed the long‐term study. Based on their seizure diaries, there was lack of efficacy in five out of 10 people in our study. The other three people who exited the study early showed efficacy in their seizure diaries. In our opinion, these data and events illustrate the various complications when treating adults with refractory epilepsy and are not surprising. Taken together, the study showed that only few adults with epilepsy followed the full 48‐week long‐term add‐on treatment with triheptanoin, although there were few side effects. This is similar to findings in several children with refractory epilepsy, where 8 out of 12 children tolerated add‐on UX007 treatment for 0.25‐2.5 years.^27^

The low number of treatment‐related side effects in this study was expected, as the participants were already experienced from the previous randomized study with MCT or triheptanoin add‐on treatment and how to avoid gastrointestinal side effects. Thus, it is likely that only patients, who in general tolerate addition of oil to their diet well, enrolled into the open‐label extension study. We were aware that body weight gain would most likely be an issue, which we tried to avoid by mandatory dietitian counseling. Except for one person, body weight changes were not of clinical significance. On average, participants’ body weight increased by 2 kg, similar to that seen after 12‐week treatment in the previous MCT vs triheptanoin study. In most people, body weight stabilized after an initial increase, indicating that with dietitian advice long‐term weight gain is not a common constrain of this treatment.

### 4.2 Efficacy, limitations, generalizability, and interpretation of antiseizure effects

While this was an open‐label study in only 10 adults with epilepsy and drug‐resistant seizures (excluding the one patient who was diagnosed as having psychogenic nonepileptic seizures during the trial), five people experienced >50% reductions in seizure frequency from baseline, with one patient being rendered completely seizure‐free for 24 weeks. All of these 5 patients had drug‐resistant focal seizures and three of them showed fewer seizures with MCT or triheptanoin in the previous trial and triheptanoin in this trial. These responsive patients may be those in whom local energy shortages can contribute to their seizures, and MCTs or triheptanoin provides extra energy to prevent seizure generation according to our metabolic hypothesis.^5^, ^6^ This is corroborated by the finding that five participants did not have any clinically significant reductions in seizure frequency in both the previous and this trial and indicates that only certain types of epilepsy respond to treatment with even or uneven MCTs.

The results of the previous open‐label trial of UX007 (triheptanoin) in children with treatment‐resistant epilepsy were similar to this trial in adults, with 5 out of the 8 children who tolerated UX007 treatment showing a >50% reduction in seizure frequency, with one child seizure‐free for 30 weeks. The reductions in seizure frequency in five out of 10 participants treated with UX007 are encouraging and are similar to other types of dietary treatments reported in adults. In a recent meta‐analysis, 53% of adults completing the ketogenic and Atkins diet studies showed >50% and 13% more than 90%, reduction in seizure frequencies.^33^ The results of this and our previous triheptanoin trial open up new possible treatment options for patients with drug‐resistant epilepsy. Opposite to ketogenic and modified Atkins diets, additions of triheptanoin or MCTs do not require strict avoidance of certain macronutrients like carbohydrates. This is expected to increase compliance with triheptanoin over these strict dietary treatments. This is especially important for adults, who have more difficulties to follow strict dietary regimen compared to children. However, the low long‐term retention rate in this study is of potential concern, but larger studies are needed and the oil formulation could be potentially improved by powdering.

On the other hand, in the previous randomized blinded MCT vs triheptanoin trial in adults only one out of 9 people had >50% reduction in seizure frequency, while 5 out of 11 adults showed efficacy with MCT. We did not target a specific type of seizure or epilepsy and the number of patients enrolled was small. Thus, the extent  to which triheptanoin is truly effective and/or different to MCT treatment regarding seizure prevention remains to be established. Biochemically, triheptanoin appears to be superior due to its anaplerotic properties; however, both treatments can provide the TCA cycle with acetyl‐CoA^5^ and MCTs are already commercially available.

In addition, in the future it will be important to avoid body weight gain with triheptanoin or MCT in people with epilepsy. We propose to combine the treatments with a low glycemic index diet.

## 5 CONCLUSIONS AND FUTURE DIRECTIONS

This is the first long‐term open‐label trial of add‐on UX007 (triheptanoin) treatment for drug‐resistant epilepsy in adults. The efficacy observed in five out of ten patients evaluated, all with focal seizures, raises the hope that metabolic therapy with triheptanoin could be a useful approach to control seizures in patients with drug‐resistant focal epilepsy. However, the low retention rate with the long‐term add‐on treatment (2 out of 10 adults) is of potential concern, although there were few side effects. Improved formulations and lower dosages may be needed to improve long‐term compliance. Larger studies are necessary to confirm these antiseizure effects in adults and to characterize the needed dosages and responsive types of seizures and epilepsy.

## CONFLICT OF INTEREST

Ultragenyx Pharmaceuticals Inc provided UX007 and the funding for the study. Dr Borges received a license fee payment and research support from Ultragenyx Pharmaceutical for other studies. Dr Terence J O’Brien reports grants and personal fees from UCB Pharma, Eisai, and Zynerba Pharmaceuticals, outside the submitted work. He has also received research grants from the NHMRC, NINDS, and the RMH Neuroscience Foundation. Dr Kwan/his institution received speaker or consultancy fees and/or research grants from Eisai, GlaxoSmithKline, Johnson & Johnson, Pfizer, and UCB Pharma. He is supported by the Medical Research Future Fund Practitioner Fellowship. He has received research grants from the National Health and Medical Research Council of Australia, the Australian Research Council, the US National Institute of Health, Hong Kong Research Grants Council, Innovation and Technology Fund, Health and Health Services Research Fund, and Health and Medical Research Fund.

## Supporting information

Fig S1

Fig S2

Fig S3

Fig S4

## Data Availability

The corresponding author has access to all data, which will be made available upon request.
