# Supplementary figures and images for: Open‐label long‐term treatment of add‐on triheptanoin in adults with drug‐resistant epilepsy
Source: Epilepsia Open. 2020 Apr 12;5(2):230–9. doi: 10.1002/epi4.12391 (PMC7278596; doi:10.1002/epi4.12391)

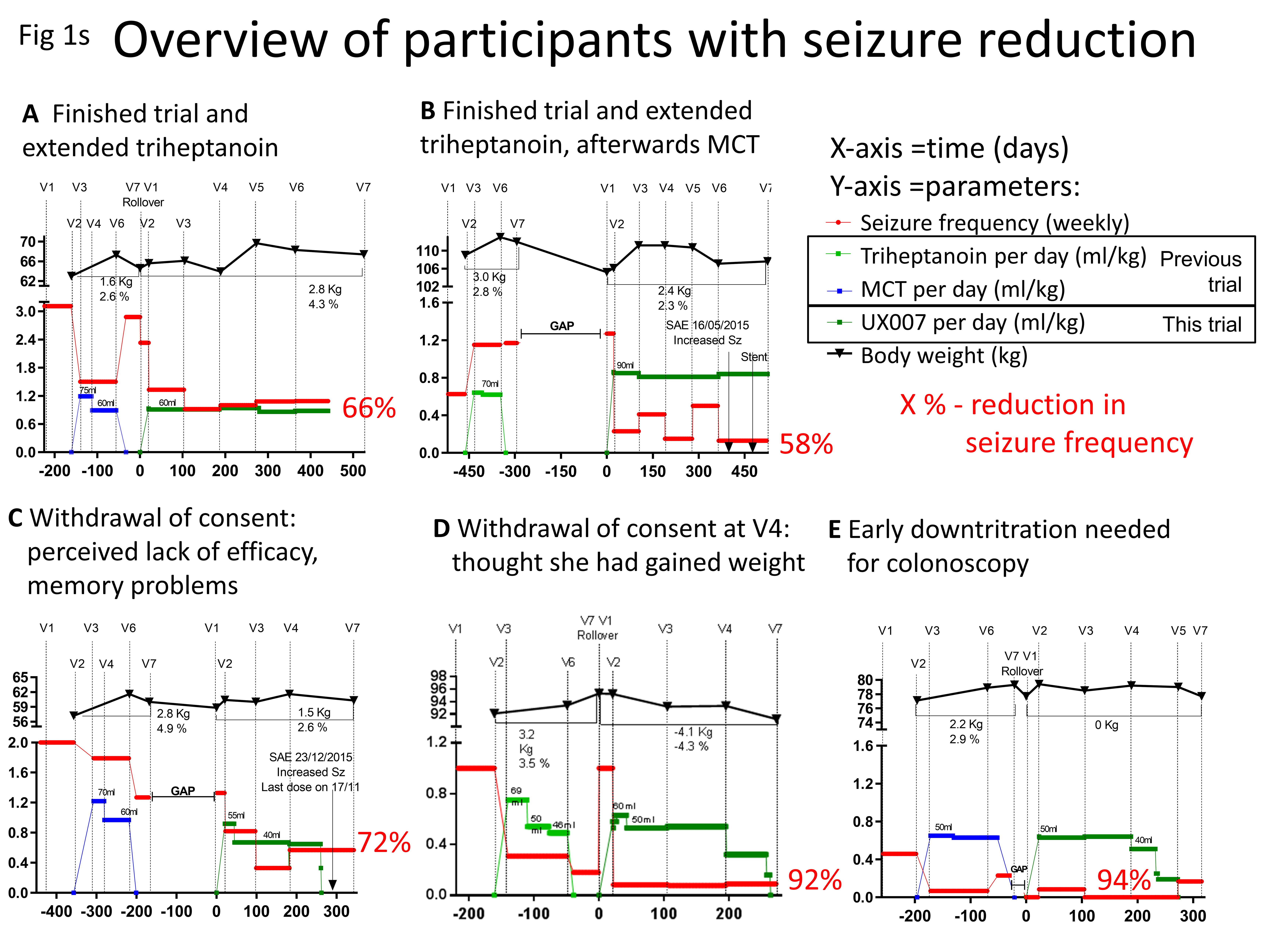

Supplement: Supplementary file 1 — Fig S1 [file EPI4-5-230-s001.tif]

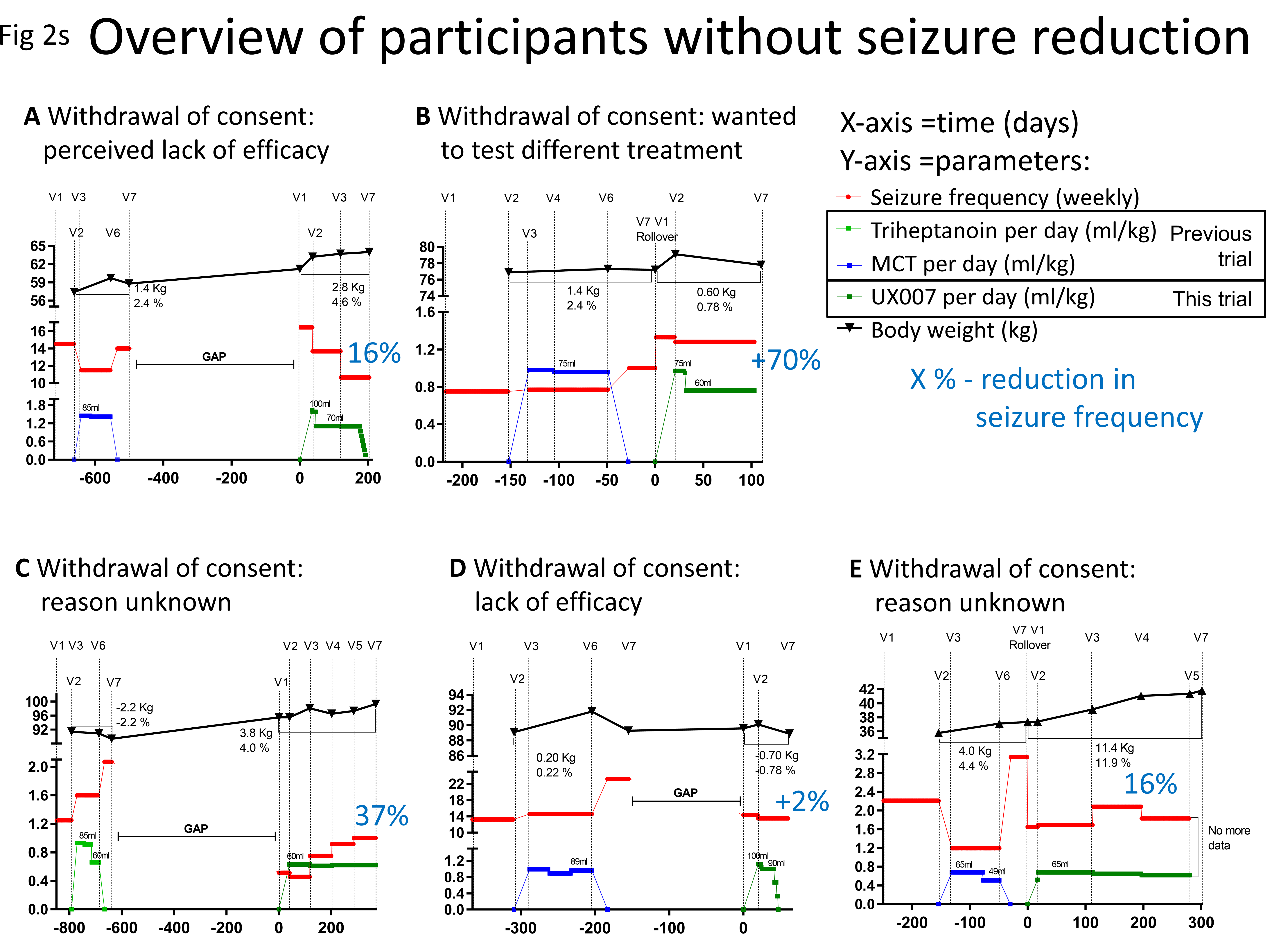

Supplement: Supplementary file 2 — Fig S2 [file EPI4-5-230-s002.tif]

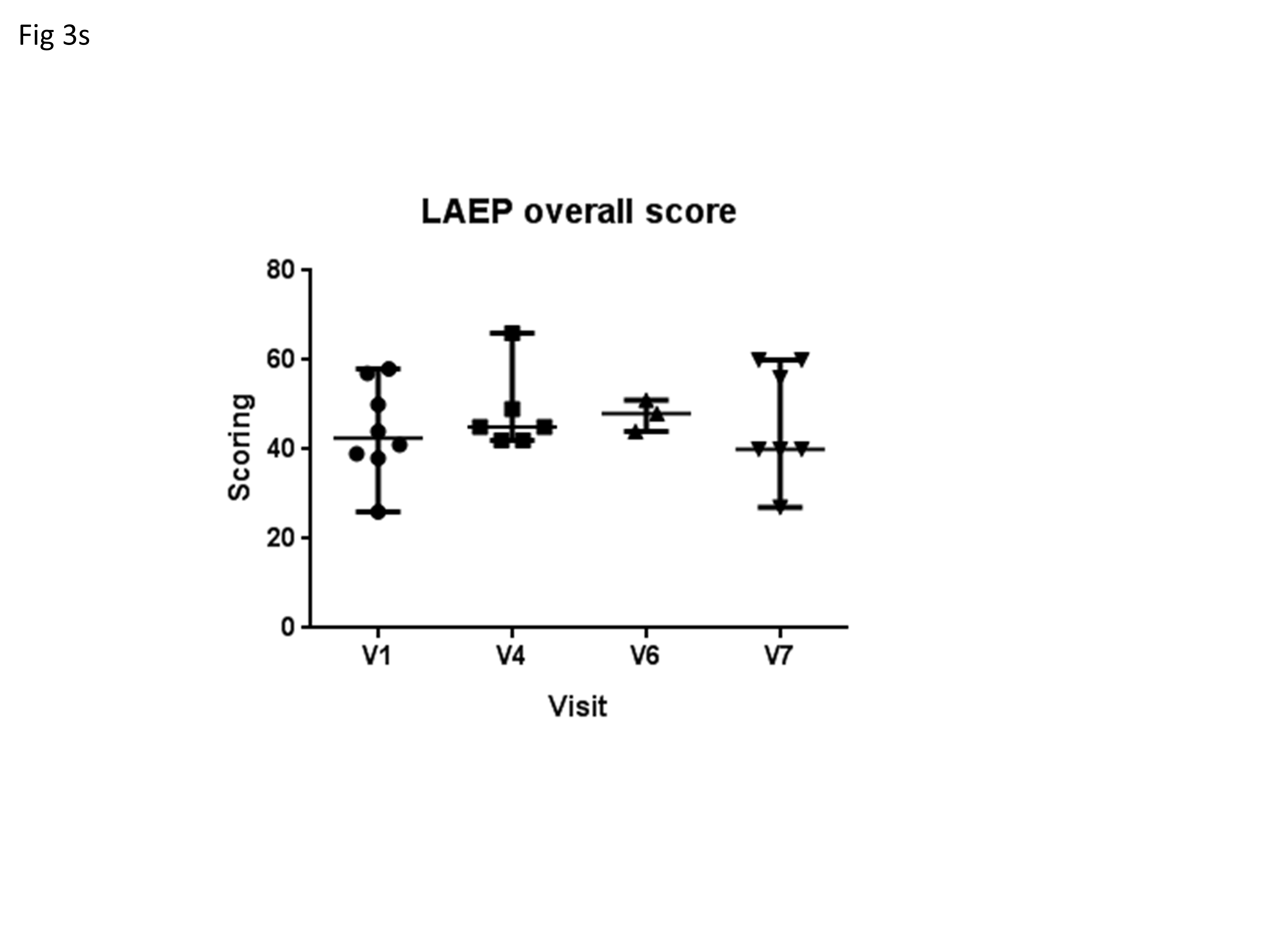

Supplement: Supplementary file 3 — Fig S3 [file EPI4-5-230-s003.tif]

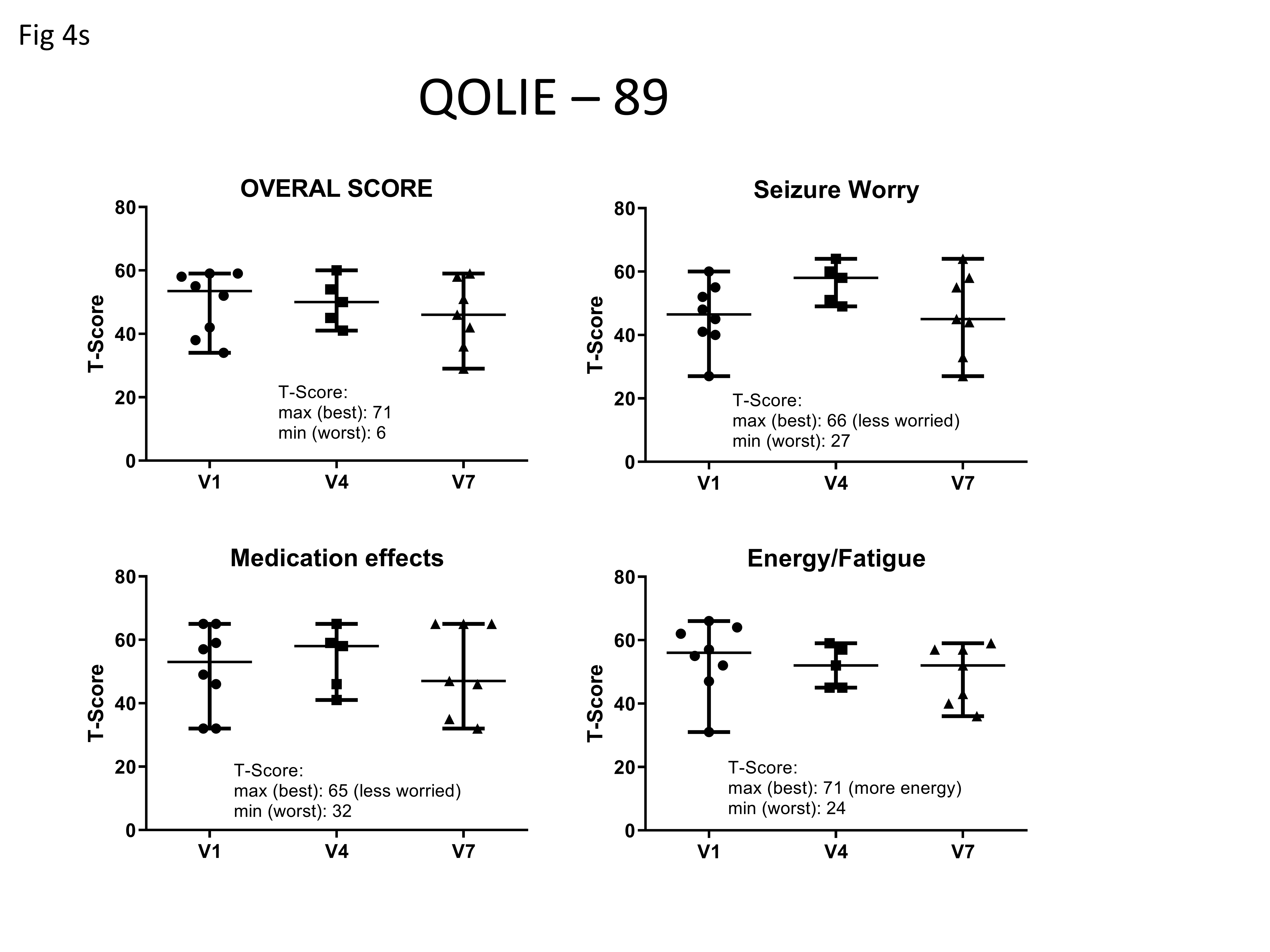

Supplement: Supplementary file 4 — Fig S4 [file EPI4-5-230-s004.tif]
